# Machine-Learning-Driven Phenotyping in Heart Failure with Preserved Ejection Fraction: Current Approaches and Future Directions

**DOI:** 10.3390/medicina61111937

**Published:** 2025-10-29

**Authors:** Victoria Potoupni, Athanasios Samaras, Christodoulos Papadopoulos, Aristi Boulmpou, Theodoros Moysiadis, Georgios Zormpas, Apostolos Tzikas, Nikolaos Fragakis, George Giannakoulas, Vassilios Vassilikos

**Affiliations:** 1 Third Department of Cardiology, Ippokratio General Hospital, Aristotle University of Thessaloniki, 546 42 Thessaloniki, Greece; 2 Second Department of Cardiology, Ippokratio General Hospital, Aristotle University of Thessaloniki, 546 42 Thessaloniki, Greece; 3 Department of Computer Science, School of Sciences and Engineering, University of Nicosia, 2417 Nicosia, Cyprus; 4 First Department of Cardiology, AHEPA University Hospital, Aristotle University of Thessaloniki, 546 36 Thessaloniki, Greece

**Keywords:** heart failure, cardiac dysfunction, HFpEF, machine learning, patient stratification, cardiac phenotyping

## Abstract

Heart failure with preserved ejection fraction (HFpEF) remains a major clinical challenge due to its heterogeneous presentation and limited therapeutic options. Accurate patient phenotyping is essential to improve diagnosis, prognostication, and treatment personalization. Machine learning (ML) has emerged as a powerful tool to identify clinically meaningful HFpEF subgroups by integrating diverse data sources, including clinical, imaging, biomarker, and physiological parameters. ML-based models can uncover subtle patterns not captured by traditional methods, offering improved risk stratification, earlier intervention, and guidance toward individualized therapy. Future progress will rely on standardized data collection, validation across populations, and incorporation into clinical decision support systems. Advancements in explainable artificial intelligence, federated learning, and multi-omics integration are expected to further refine phenotyping strategies and translate into improved patient outcomes. Continued interdisciplinary collaboration is essential to unlock the full potential of ML in transforming HFpEF management.

## 1. Introduction

Heart failure with preserved ejection fraction (HFpEF) poses a significant challenge to healthcare systems worldwide due to its rising prevalence and high associated morbidity and mortality [1]. Impaired left ventricular (LV) relaxation and/or increased stiffness represent the hallmark of HFpEF, leading to diastolic dysfunction and symptoms of heart failure (HF) despite preserved systolic function [2]. The resulting elevation in filling pressures contributes to pulmonary congestion and a broad spectrum of clinical manifestations [3]. In contrast to heart failure with reduced ejection fraction (HFrEF), where several therapeutic strategies have demonstrated substantial benefit, effective treatment options for HFpEF remain elusive. This underscores the urgent need for deeper pathophysiological understanding and the development of more targeted treatment approaches.

**Figure 1:**
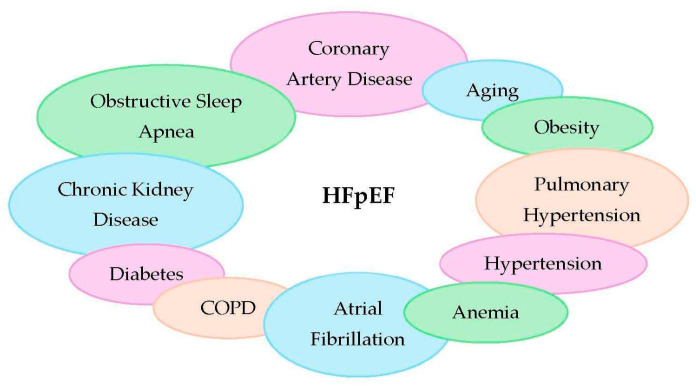
Comorbidities in HFpEF.

Extracardiac comorbidities are highly prevalent in HFpEF and often precede its clinical stage (Figure 1) [4]. This observation supports the view that HFpEF is not a single entity but rather an heterogenous syndrome encompassing distinct patient subgroups. Consequently, clinical phenotyping has become a key strategy to better understand disease heterogeneity, pathophysiology, and patterns of clinical presentation, and test tailored therapeutic interventions.

In this context, machine learning (ML) has shown great promise as a tool for HFpEF phenotyping, enabling data-driven identification of distinct patient subgroups based on clinical, imaging, biomarker, and physiological information. ML approaches are well-suited for recognizing complex, non-linear patterns in large and multidimensional datasets that might be imperceptible to conventional statistical techniques. Recent studies have demonstrated the potential of ML-based phenomapping to reveal mechanistically and prognostically distinct HFpEF phenotypes, providing important insights into the disease and optimizing therapeutic strategies [5,6,7,8,9,10].

A multifaceted management approach that integrates genetic, biomarker, and clinical data, alongside patient preferences, is increasingly recognized as the cornerstone of personalized care in HFpEF. The parallel development of novel pharmacological and device-based therapies within this framework sets the stage for a new era in HFpEF management. However, the full realization of this vision requires coordinated research efforts, including well-designed clinical trials that address phenotypic diversity and the advancement of robust tools for precise patient stratification.

The goal of this review is to provide a comprehensive overview of clinical phenotyping in HFpEF using ML approaches, with emphasis on the pathophysiological insights gained and the ways in which these methods may inform therapeutic algorithms. In this context, the manuscript uniquely contributes by integrating emerging methodologies, including federated learning to enable multi-center data collaboration, explainable AI to enhance interpretability of complex predictive models, deep learning to capture complex non-linear relationships within high-dimensional data, and multi-omics analyses, thereby providing a comprehensive framework for improved patient stratification.

## 2. Challenges of Traditional Methods in HFpEF Phenotyping

Accurate phenotyping is essential for diagnosis, risk stratification, and the development of targeted therapies in HFpEF. However, several barriers continue to complicate this process, ranging from limitations of traditional diagnostic methods to emerging clinical challenges unique to ML-based approaches [5].

Traditional phenotyping strategies in HFpEF combine clinical assessment, imaging, biomarkers, and invasive hemodynamics, but each has important shortcomings. Clinical assessment remains central but is inherently subjective, as the symptoms of HFpEF often overlap with those of other cardiovascular, respiratory, or systemic disorders. Echocardiography, the cornerstone imaging modality, provides valuable information but is highly operator-dependent and subject to interobserver variability. Although advanced techniques such as cardiovascular magnetic resonance (CMR) can reveal detailed myocardial tissue characteristics and fibrosis, their high cost and limited availability preclude routine use [6].

Biomarkers hold a promising role in HFpEF phenotyping, and natriuretic peptides remain the only markers with consistent diagnostic utility in clinical practice. Novel molecules such as galectin-3 and soluble ST2 show promise for refining phenotyping, but their clinical role is still under investigation [7]. Finally, invasive hemodynamic assessment through right heart catheterization provides the most precise information on filling pressures and pulmonary vascular load, yet it is resource-intensive, requires specialized expertise, and carries procedural risks [8].

Recent advancements in ML have shown considerable promise in addressing the shortcomings of traditional HFpEF phenotyping methods. ML algorithms can process large, multidimensional datasets from diverse sources, including clinical profiles, cardiac imaging, and biomarker measurements [9]. By capturing subtle and complex patterns, these models offer new opportunities to improve diagnostic accuracy and patient stratification. Nevertheless, ML-based phenotyping is not without obstacles. Data quality and standardization remain critical issues, as heterogeneous and incomplete inputs can compromise performance. Effective integration of data from multiple modalities requires sophisticated algorithms capable of handling varied structures and formats. Equally important is the question of interpretability: for ML to gain traction in clinical practice, physicians must be able to understand the rationale behind a model’s predictions, ensuring transparency and building trust in its application [10].

## 3. Machine Learning Techniques in HFpEF Phenotyping

ML offers a range of techniques that can be applied to HFpEF phenotyping, each with distinct advantages and limitations (Table 1). These approaches can broadly be divided into supervised learning, unsupervised learning, and deep learning methods.

Supervised learning relies on labeled datasets in which outcomes are already known. In HFpEF, algorithms such as logistic regression, decision trees, random forests, and support vector machines (SVMs) have been used to distinguish patients from those with other types of HF and to identify meaningful subgroups [11,12]. Logistic regression is applied to evaluate the relationship between one or more independent (predictor) variables and a binary dependent (outcome) variable [13]. While simple and highly interpretable, is often limited in capturing the non-linear interactions typical of HFpEF datasets. A decision tree is a structured predictive model that represents the process of computing the output of a function f(x) through a sequence of hierarchical decision rules applied to the input variables [14]. Decision trees generate transparent, rule-based models, but they are prone to overfitting. Random forests, by combining multiple trees, mitigate this weakness and improve accuracy, though at the cost of interpretability. Random forests have been employed to predict mortality and hospitalization outcomes in patients with HFpEF [15]. SVMs have been used to evaluate the prognostic significance of multiple circulating biomarkers in patients with acute HFpEF [16]. They are powerful in handling high-dimensional data and non-linear relationships, yet they are sensitive to parameter tuning and are often criticized for their ‘black-box’ nature [17].

Unsupervised learning does not depend on predefined outcomes and is particularly valuable for uncovering hidden phenotypes. Clustering techniques, including k-means, hierarchical clustering, and Gaussian mixture models (GMMs), have been used to classify HFpEF patients into subgroups based on shared clinical and imaging features. These methods have revealed phenotypes with distinct prognoses and therapeutic responses [18,19,20,21,22]. Hierarchical clustering is especially useful for exploring the nested relationships between subgroups, while GMMs provide a probabilistic framework that accounts for overlap between phenotypes [12]. However, these models are highly sensitive to data preprocessing, variable selection, and assumptions about data distribution.

Deep learning, a subset of ML based on neural networks, has shown significant promise in HFpEF phenotyping due to its capacity to analyze large and complex datasets. Convolutional neural networks (CNNs) have been applied to echocardiographic images, extracting subtle features that may be overlooked by traditional analysis [12]. Recurrent neural networks (RNNs), designed to handle sequential data, have been used to assess time-series information from wearable devices and electronic health records (EHRs), providing insights into disease progression and phenotyping variation. Autoencoders, another deep learning approach, can reduce the dimensionality of high-dimensional data and identify latent features associated with HFpEF phenotypes [12]. While deep learning excels in capturing complexity, it is limited by issues of interpretability and carries a heightened risk of overfitting, particularly when datasets are small or noisy.

A critical aspect of applying ML on HFpEF phenotyping is feature selection and engineering. Identifying the most informative variables among clinical, imaging, and biomarker data improves model accuracy and robustness. Techniques such as recursive feature elimination and LASSO (Least Absolute Shrinkage and Selection Operator) regression have been applied successfully, while feature engineering can further enhance model performance by generating novel variables from existing data. Poor feature selection, however, risks bias and overfitting, especially when dealing with incomplete or imbalanced datasets.

The effective use of ML in HFpEF phenotyping depends not only on the choice of algorithm but also on careful data integration, robust validation, and interpretability. Increasingly, explainability methods such as SHAP (Shapley Additive Explanations) values and LIME (Local Interpretable Model-agnostic Explanations) are being incorporated to clarify how individual features influence predictions, thereby improving transparency and clinician confidence [23]. A recent example is the development of a diagnostic model based on electronic health records, which used SHAP values to highlight the clinical features driving predictions. The model achieved high accuracy in distinguishing HFpEF from both non-cardiac dyspnea and HFrEF, demonstrating the potential of interpretable ML for clinical decision-making [24].

## 4. Major Machine Learning-Based Phenotypes

Phenotype 1: Inflammation-driven HFpEF

One of the identified phenotypes, as stated by Galli et al. (2021) [25], was characterized by high levels of systemic inflammation. Patients in this subgroup exhibited elevated levels of inflammatory biomarkers such as C-reactive protein (CRP) and interleukin-6 (IL-6). This phenotype was also associated with high prevalence of comorbid conditions, such as obesity and diabetes mellitus, which have been proven to contribute to systemic inflammation [25].

According to the pathophysiology driving this specific phenotype, inflammation holds a central role in the development and progression of HFpEF. The inflammatory process is closely related to endothelial dysfunction, myocardial fibrosis, and increased myocardial stiffness, contributing to elevated cardiac filling pressures. Patients with an inflammation-driven phenotype tend to demonstrate worse clinical outcomes, including increased risk of recurrent hospitalization and higher mortality. Anti-inflammatory therapies such as statins or specific anti-inflammatory agents may be beneficial in controlling systemic inflammation and improving clinical outcomes among this distinct HFpEF subgroup [25].

Phenotype 2: Fibrosis-driven HFpEF

Another identified HFpEF phenotype was characterized by pronounced myocardial fibrosis [25]. This subgroup is characterized by elevated levels of biomarkers indicative of fibrosis, such as galectin-3 and soluble ST2, while imaging studies, including CMR, revealed extensive late gadolinium enhancement. Patients categorized in this phenotype often present with a history of hypertensive heart disease, contributing to increased LV mass and excess myocardial fibrosis. Pharmaceutical treatments targeting fibrosis, such as mineralocorticoid receptor antagonists (MRAs) or emerging anti-fibrotic drugs like pirfenidone, may be beneficial for this patient population. Managing hypertension aggressively and controlling underlying comorbidities are also critical to reduce myocardial stress and progression of fibrosis [25].

Phenotype 3: Volume overload HFpEF

Shah et al. conducted a phenomapping study using unsupervised ML techniques to analyze clinical, echocardiographic, and biochemical data from HFpEF patients [9]. They identified a phenotype characterized by signs of volume overload, as patients in this subgroup frequently presented with elevated natriuretic peptides, markers of increased cardiac wall stress due to volume overload. This phenotype also presented a higher prevalence of atrial fibrillation (AF), contributing to hemodynamic instability and volume overload [9]. The pathophysiological mechanism behind this phenotype comprises excessive fluid retention and impaired renal function, leading to increased intravascular volume and elevated LV filling pressures. Management focuses predominantly on optimizing diuretic therapy to control fluid status and prevent HF exacerbations.

Phenotype 4: Right Ventricular Dysfunction

A HFpEF patient subgroup characterized by significant right ventricular (RV) dysfunction was identified by Shah et al., who stated that this phenotype exhibited elevated right-sided pressures, assessed either through echocardiography or right heart catheterization, and symptoms consistent with right HF. Elevated biomarkers indicative of RV dysfunction, such as NT-proBNP, were also common in this group [19]. The underlying pathophysiology in this phenotype is likely related to pulmonary hypertension and RV overload, which can occur secondary to left-sided HF or as a primary disease. The inability of the RV to handle increased afterload leads to symptoms of systemic congestion, such as peripheral edema and ascites. Management includes pharmaceutical regimens reducing pulmonary pressures, such as phosphodiesterase-5 inhibitors or endothelin receptor antagonists, in distinct cases. Patients may also benefit from cardiac rehabilitation with an eye to improve functional capacity and health-related quality of life [19].

Phenotype 5: Exercise-Induced HFpEF

Another distinct HFpEF phenotype involves patients who predominantly experience symptoms during physical exertion. This exercise-induced HFpEF phenotype was characterized by normal resting hemodynamics but significant abnormalities during stress testing. Such patients often demonstrate an exaggerated increase in filling pressures and pulmonary pressures during exercise, indicative of inability to augment cardiac output appropriately [25]. Management focuses on improving exercise tolerance and cardiac output during physical activity. Structured exercise programs, potentially combined with medication such as beta-blockers or ivabradine to control heart rate, may enhance exercise capacity and reduce symptoms [5].

Representative ML-based phenotyping studies

Several studies have implemented unsupervised ML to identify distinct HFpEF subtypes, revealing the large biological and clinical heterogeneity of the syndrome. However, despite consistent use of clustering techniques, these studies vary significantly in their data sources, populations, and cluster characteristics, raising questions about reproducibility and generalizability.

Shah et al. (2015) [9] utilized unsupervised ML, specifically phenomapping, to classify HFpEF patients into 3 distinct phenogroups; one group was characterized by obesity and high prevalence of metabolic syndrome, another group by higher AF incidence, and a third group with high levels of inflammatory markers and myocardial fibrosis [9]. This seminal study pioneered phenotype discovery in HFpEF but was limited by a single-center cohort and lack of external validation. Kyodo et al. also (2023) [11] applied unsupervised ML with a variational Bayesian–Gaussian mixture model (VBGMM) to classify Japanese HFpEF patients into 3 phenogroups. One phenogroup was older with a higher prevalence of comorbidities, another included younger patients with fewer comorbidities but higher levels of inflammatory markers, and the third demonstrated a higher prevalence of AF [11]. The divergence in phenotypic profiles between Shah and Kyodo’s cohorts underscores the influence of ethnic, geographic, and clinical context on unsupervised ML outcomes. It also highlights the sensitivity of clustering outputs to input features and population characteristics. Further extending the use of unsupervised learning, Pierre-Jean et al. classified HFpEF patients into 4 phenotypic clusters based on clinical variables from electronic health records and echocardiographic parameters, where each cluster exhibited distinct prognostic outcomes [26].

In the ASCEND-HF trial, hierarchical clustering and latent class analysis were applied to identify distinct clusters in hospitalized HFpEF patients. This study identified four clusters with varying demographics, comorbidities, and clinical outcomes. Mortality rates differed significantly among the clusters [27]. This large, multicenter dataset enhanced statistical power but focused on an acutely ill population, potentially skewing phenotypes compared to chronic outpatient cohorts. Carlson and colleagues used principal component analysis, k-means clustering, and hierarchical clustering on optimized parameters representing cardiovascular function to identify 3 subgroups: HFrEF-like HFpEF, classic HFpEF, and a non-consistently clustering group. This study highlighted various mechanistic differences between HFpEF groups that were not initially apparent from clinical data [28].

Biomarker-based approaches also gained traction. Woolley and colleagues performed an unsupervised cluster analysis using 363 biomarkers from 429 HFpEF patients. They identified 4 distinct subgroups with varying clinical characteristics, such as diabetes mellitus prevalence, age, body size, and NT-proBNP levels. Pathway analysis linked the biomarker profiles to specific biological processes [22]. Rabkin et al. used hierarchical clustering to identify distinct biomarker phenogroups within HFpEF patients, each characterized by unique clinical features and cardiac structural differences [29]. These studies underscore the potential of high-dimensional omics data to enhance biological interpretability of phenogroups, but challenges remain in harmonizing biomarker assays across studies and translating findings into therapeutic strategies.

Ahmad et al. highlighted the potential of various ML techniques to improve the diagnosis and treatment of HFpEF by identifying distinct phenotypes and predicting treatment responses. This study emphasized the need for high-quality data and validation in independent cohorts to ensure the robustness of ML models [12]. This reflects a broader issue in the field: while ML models excel in discovery, their utility in practice depends on rigorous external validation and model interpretability.

## 5. Tailored Therapy Based on ML Phenotyping

The integration of ML phenotyping into clinical practice has led to the development of both currently implemented and emerging therapies tailored to specific HFpEF phenotypes.

European guidelines suggest the use of diuretics and sodium-glucose cotransporter-2 (SGLT2) inhibitors in HFpEF patients [20]. Potentially promising therapies identified through ML also comprise novel agents targeting fibrosis and inflammation. For example, pirfenidone in the PIROUETTE trial and dapagliflozin have been shown to be beneficial in reducing myocardial fibrosis in patients with HFpEF [30,31,32,33]. Emerging evidence highlights the role of systemic inflammation in HFpEF and the therapeutic potential of targeting inflammatory pathways, including IL-1β and IL-6, which may reduce myocardial fibrosis, reverse LV hypertrophy, and improve cardiomyocyte function [34,35]. In addition, SGLT2 inhibitors have been shown to lower hospitalization rates in HFpEF patients, exerting anti-inflammatory and cardioprotective effects through multiple mechanisms, including metabolic improvements, reduction in oxidative stress, modulation of cytokine release, and ketone-mediated inhibition of histone deacetylases and NLRP3 inflammasomes [36,37]. The DELIVER and EMPEROR-Preserved trials demonstrated that dapagliflozin and empagliflozin significantly reduced the risk of worsening heart failure or cardiovascular mortality in patients with mildly reduced or preserved ejection fraction [38,39].

AF commonly coexists with HFpEF, leading to worsened symptoms and clinical outcomes. Catheter ablation has emerged as a promising treatment option in this group, offering effective rhythm control, reduced AF recurrence, and potential enhancements in functional status and health-related quality of life. However, the effectiveness of ablation in HFpEF patients may be more variable than in those with HFrEF, highlighting the importance of individualized patient assessment [40]. ML-based identification of patients with volume overload or right ventricular dysfunction can guide enrollment in studies testing optimized diuretic regimens or pulmonary vasodilators. Exercise-induced HFpEF phenotypes, on the other hand, could inform trials investigating exercise training protocols or chronotropic modulation therapies.

## 6. Advancements in ML Techniques

Emerging technologies and methodologies are continuously advancing the field of ML phenotyping in HFpEF. The development of explainable AI (XAI) techniques belongs among the key advances in ML; XAI aims to make ML models more transparent and interpretable, allowing clinicians to understand the rationale behind model predictions. Techniques such as SHAP (Shapley Additive Explanations) values and LIME (Local Interpretable Model-agnostic Explanations) may shed light on the contribution of individual features to model outputs [10].

In the same frame, federated learning is an emerging approach that allows ML models to be trained on decentralized data sources while preserving data privacy. In the context of HFpEF, federated learning can enable the development of robust phenotyping models using data from multiple institutions without the need to share sensitive patient information [41]. Advancements in multi-omics technologies, including genomics, proteomics, and metabolomics, provide an opportunity to integrate comprehensive molecular data into ML phenotyping. By analyzing multi-omics data alongside clinical and imaging information, ML models can uncover deeper insights into the molecular mechanisms underlying HFpEF and identify novel therapeutic targets [42].

Deep learning and neural networks continue to advance, offering powerful tools for complex data analysis. CNNs are particularly effective in analyzing imaging data derived from various diagnostic modalities, such as echocardiography and CMR, to identify phenotypic patterns. RNNs and long short-term memory (LSTM) networks are also valuable for analyzing time-series data from wearable devices and continuous monitoring systems [43].

## 7. Future Directions and Areas of Improvement

The literature provides growing evidence on the transformative potential of artificial intelligence in cardiology and electrophysiology, highlighting its emerging role in clinical decision-making, procedural guidance, and risk stratification. Recent discussions, such as that by Tsampasian et al., further emphasize how large language models and machine learning tools could reshape cardiovascular care by enhancing diagnostic precision and workflow efficiency [44]. Nonetheless, these advances also bring attention to persistent barriers to clinical adoption—including data governance, regulatory compliance, and the need for transparent, explainable systems that can foster clinician trust and accountability. To fully realize the potential of ML phenotyping in HFpEF, future research should focus on several key areas. Future research should prioritize the validation of ML phenotyping algorithms using large, diverse, and representative datasets.

Building upon the importance of validation, another critical area is the integration of real-world data. Integrating real-world data from EHRs, registries, and wearable devices can significantly improve the applicability and accuracy of ML phenotyping models. Studies demonstrate that integrating real-time ECG telemetry and sensor-derived data—such as heart rate variability and physical activity—can enhance diagnostic accuracy and enable continuous monitoring in HFpEF patients [45]. The SEISMIC-HF 1 study evaluated the feasibility and accuracy of a wearable sensor-based patch for non-invasive estimation of pulmonary capillary wedge pressure (PCWP) in patients with heart failure. The results demonstrated that the wearable could provide real-time, continuous hemodynamic monitoring with clinically acceptable accuracy [46]. To date, invasive electrophysiological parameters—such as atrial voltage mapping or conduction velocity measurements—have not been integrated into machine learning-based phenomapping approaches in HFpEF. This represents a promising and largely unexplored area for future research. For instance, atrial strain imaging via speckle tracking echocardiography [47] or MRI feature-tracking has demonstrated prognostic significance in HFpEF, and heart catheterization measurements have proven discriminative for diagnosis and risk prediction [48,49]. Standardized data collection protocols and interoperable systems may facilitate the aggregation and analysis of data [50,51].

To ensure these tools are used effectively, education and training of healthcare providers is equally important. Educating clinicians about the principles and applications of ML phenotyping is crucial for its successful integration into clinical practice. Training programs should be developed to enhance clinicians’ understanding of ML techniques, their potential benefits, and how to interpret ML-generated recommendations. Equally important is the involvement of patients in the design and application of ML phenotyping tools to ensure their effectiveness and widespread adoption. As these technologies move closer to clinical adoption, regulatory and ethical considerations must also be addressed. Regulatory bodies need to establish guidelines for the use of ML in healthcare, ensuring patient safety, data privacy, and algorithm transparency. Ethical considerations such as bias in ML models and the potential for algorithmic discrimination must also be addressed.

Building upon this need for standardized inputs, the implementation of ML-based phenotyping within Clinical Decision Support Systems (CDSSs) represents a practical application of these efforts. CDSSs can help clinicians make informed decisions at the point of care by providing real-time recommendations based on ML-derived insights, such as identifying patient subgroups and suggesting tailored therapies. Ultimately, ML phenotyping can enable the development of personalized treatment plans tailored to the specific pathophysiological mechanisms underlying each patient’s HFpEF [52]. It can improve diagnostic accuracy by identifying subtle patterns and biomarkers that may be missed by traditional methods; this approach could have an even greater impact in primary prevention settings.

## 8. Conclusions and Limitations

HFpEF is a complex and heterogeneous condition that presents significant challenges in diagnosis and treatment. Current therapeutic approaches have limited efficacy due to the varied phenotypes of the disease, highlighting the need for personalized medicine. ML offers a promising avenue for advancing HFpEF phenotyping by analyzing large and diverse datasets to identify novel patient subgroups.

While ML presents a promising tool for phenotyping HFpEF, several limitations must be acknowledged. One major challenge is the quality and consistency of data, as ML models require standardized, high-quality datasets to perform optimally, yet clinical data are often incomplete or heterogeneous. The integration of multimodal data, including clinical assessments, imaging, biomarkers, and hemodynamic measurements, adds complexity, as combining these disparate data types requires sophisticated techniques. In addition to these methodological barriers, there are substantial practical obstacles to clinical implementation. The lack of interoperability between hospital information systems hinders large-scale data integration, while regulatory and ethical constraints surrounding patient privacy and cross-institutional data sharing further limit collaborative efforts. Moreover, the interpretability of ML models, particularly deep learning-based architectures, remains a key source of skepticism among clinicians, as black-box algorithms often fail to provide transparent, explainable decision pathways essential for clinical acceptance. Generalizability is another major concern, as models developed using specific cohorts may not perform as well in diverse populations.

Ethical considerations, including issues of data privacy, security, and potential biases in the training data, pose additional risks, particularly regarding equity in healthcare outcomes. Lastly, many ML models lack extensive clinical validation, and their utility in real-world practice remains uncertain without prospective trials and rigorous validation efforts. Addressing these limitations is crucial for fully harnessing the potential of ML in advancing HFpEF phenotyping and personalized treatment strategies.

## Figures and Tables

**Table 1:** Comparative summary table outlining machine learning techniques.

Technique	Interpretability	Handles Complexity	Application in HFpEF	Advantages	Limitations
Logistic regression	High	Low–Moderate	Classifying HFpEF vs. other HF types using clinical predictors	Simple, interpretable, well-understood	May underperform with complex/non-linear relationships
Decision trees	High	Moderate	Clear rule-based HFpEF classification	Easy to interpret, generates clear rules	Prone to overfitting
Random forests	Moderate	High	Identifying HFpEF subgroups via ensemble decision trees	Robust, handles non-linearities, reduces overfitting	Less interpretable than single trees
Support vector machines (SVMs)	Low	High	Classifying HFpEF based on complex feature interactions	Effective in high-dimensional spaces, handles non-linearity well	Poor interpretability, sensitive to parameter tuning
k-means clustering	Moderate	Moderate	Grouping patients with similar phenotypes	Simple, fast, intuitive clustering	Assumes spherical clusters, sensitive to initialization
Hierarchical clustering	High	Moderate	Creating tree-structured patient subgroup hierarchies	Reveals subgroup relationships, no need to predefine number of clusters	Computationally expensive for large datasets
Gaussian mixture models (GMMs)	Moderate	High	Probabilistic clustering of overlapping HFpEF phenotypes	Captures uncertainty and soft clustering	Assumes Gaussian distribution, may converge to local minima
CNNs (deep learning)	Low	Very High	Extracting features from echocardiographic images for phenotype classification	Automatic feature extraction, high accuracy in image tasks	Requires large datasets, low interpretability
RNNs (deep learning)	Low	Very High	Analyzing time-series data from EHRs and wearables	Captures temporal dynamics, good for sequential data	Training complexity, vanishing gradient issues (mitigated by LSTM/GRU)
Autoencoders	Low	High	Dimensionality reduction and latent phenotype discovery	Identifies hidden patterns, reduces noise	Requires tuning, difficult to interpret latent features

## Data Availability

No new data were created or analyzed in this study.

## Abbreviations

The following abbreviations are used in this manuscript:

AI	Artificial intelligence
ARB	Angiotensin receptor blocker
ARNI	Angiotensin receptor/neprilysin inhibitor
BNP	B-type natriuretic peptide
CDSS	Clinical decision support system
CMR	Cardiac magnetic resonance
CNN	Convolutional neural network
COPD	Chronic obstructive pulmonary disease
CRP	C-reactive protein
EHR	Electronic health record
GDF-15	Growth differentiation factor-15
GMM	Gaussian mixture model
HF	Heart failure
HFpEF	Heart failure with preserved ejection fraction
HFrEF	Heart failure with reduced ejection fraction
IL-6	Interleukin-6
LSTM	Long Short-Term Memory
LV	Left Ventricle
LVEF	Left Ventricular Ejection Fraction
LVH	Left Ventricular Hypertrophy
ML	Machine Learning
MRA	Mineralocorticoid Receptor Antagonist
nt-proBNP	N-terminal pro-BNP
RAAS	Renin-Angiotensin-Aldosterone System
RNN	Recurrent Neural Network
RV	Right Ventricle
SGLT2	Sodium-Glucose Cotransporter-2
SVM	Support Vector Machine
T2DM	Type 2 Diabetes Mellitus
VBGMM	Variational Bayesian–Gaussian Mixture Model
